# HFpEF risk assessment using H_2_FPEF score in community-dwelling young Hispanic adults

**DOI:** 10.3389/fcvm.2026.1832145

**Published:** 2026-05-29

**Authors:** Andrew Kim, Fadi I. Musfee, Soumya Patnaik, Miryoung Lee, Emma Molina, David D. McPherson, Joseph B. McCormick, Susan P. Fisher-Hoch, Susan T. Laing

**Affiliations:** 1 Division of Cardiology, Department of Internal Medicine, McGovern Medical School, University of Texas Health Science Center at Houston, Houston, TX, United States; 2 Department of Epidemiology, School of Public Health, University of Texas Health Science Center at Houston, Brownsville, TX, United States

**Keywords:** cardiometabolic risk (CMR), H_2_FPEF score, heart failure with preserved ejection fraction (HFpEF), metabolic syndrome, young Hispanics

## Abstract

Heart failure with preserved ejection fraction (HFpEF) prevalence has increased with the increase in cardiometabolic risk factors and yet, diagnosis remains underrecognized in the community, with concomitant undertreatment. H_2_FPEF scoring is a validated tool for dyspnea evaluation but has potential to identify individuals with increased HFpEF risk in asymptomatic populations. Hispanics are known to have high prevalence of cardiometabolic risk, which predispose them to HFpEF. This study aimed to apply the H_2_FPEF score to a community-dwelling Hispanic cohort, and define demographic and clinical covariate associations, particularly in asymptomatic young Hispanic adults.

**Methods:**

Demographic, cardiometabolic and echocardiographic data were obtained in 1,285 asymptomatic participants (66% females, mean age 53 ± 15 years) from the Cameron County Hispanic Cohort. H_2_FPEF scoring was performed, and the sample divided into low risk (0–2 points) or intermediate-high risk (3–7 points). Analysis was dichotomized to those <60 years and ≥60 years of age, and multivariate logistic regression analyses were conducted.

**Results:**

Intermediate-high risk H_2_FPEF score was seen in 43% (*n* = 555) of the sample. Among those <60 years of age, a third (33%) already had intermediate-high H_2_FPEF scores. After adjusting for covariates, among young Hispanics, age [OR=1.02 (1.01–1.04)], metabolic syndrome [OR=2.16 (1.54–3.04)], higher hemoglobin A1c [OR 1.23 (1.08–1.39)] and sedentary lifestyle [OR=0.60 (0.41–0.88)] remained significantly associated with odds of increased H_2_FPEF score.

**Discussion:**

We showed that a large proportion of Hispanics in our cohort have intermediate-high H_2_FPEF scores, and this pattern was already seen among young Hispanics. A simple and useful tool such as the H_2_FPEF score to identify persons at risk for HFpEF, particularly in communities with limited resources, may be relevant for prevention and early intervention, specifically targeting cardiometabolic risk control in young Hispanics.

## Introduction

Heart failure with preserved ejection fraction (HFpEF) is increasingly recognized as a leading cause of heart failure-related hospitalization and death in the United States (1). The variability in reported prevalence of HFpEF relate partly to different definitions of HFpEF used, different age cut-offs in studies, as well as geographical and true ethnic variations (2, 3). Nevertheless, morbidity and mortality from HFpEF is projected to surpass that of heart failure with reduced ejection fraction, with an estimated overall lifetime healthcare cost of $126,819 per patient (2). Unfortunately, HFpEF is often underdiagnosed particularly in the community setting, reflecting the lack of consensus diagnostic criteria, its heterogeneity, and overlap with other disease conditions in both risk factors and clinical presentation.

The H_2_FPEF score is a clinically validated tool first described in a 2018 Mayo Clinic study which aggregates both clinical features and echocardiographic parameters to aid in the diagnosis of HFpEF in the setting of undifferentiated dyspnea (4). Since then, several investigators have expanded the use of this algorithm and applied the H_2_FPEF score to help ascertain incident HFpEF risk in the outpatient setting. We and others have shown that Hispanics/Latinos have a high prevalence of cardiometabolic diseases including hypertension and overweight/obesity (5, 6) which place them at increased lifetime risk for HFpEF. Furthermore, Hispanic patients hospitalized with HF were significantly younger than non-Hispanic whites, and among those hospitalized for HFpEF, more than a third of Hispanic patients (38.3%) were younger than 66 years (7). Our study applied the H_2_FPEF score to the Cameron County Hispanic Cohort (CCHC), an ongoing study of community-dwelling homogeneous cohort of Mexican-Americans who have a high prevalence of cardiometabolic risk factors (5). We aimed to determine the demographic and clinical associations of higher H_2_FPEF scores in Hispanic adults, and specifically among young Hispanics. Identifying and addressing specific cardiometabolic risk factors among Hispanics may provide a focused target for prevention of overt HF symptoms and incident HF hospitalization in this high risk disparity cohort.

## Methods

### Study population and sample

The study subjects were a subset of the Cameron County Hispanic Cohort (CCHC) who had a transthoracic echocardiogram performed as part of their routine follow up visits between 2012 and 2023. A detailed description of CCHC has been published elsewhere (5, 8). Briefly, the CCHC is a longitudinal homogenous community-dwelling Mexican American cohort living in Brownsville, Texas, located on the lower Rio Grande River at the United States-Mexico border. Study participants were recruited from randomly selected household blocks according to the 2000 Census and stratified by income as described previously (9). Inclusion criteria for the analysis are 1) self-reported Hispanic/Latino of Mexican heritage, 2) without reported cardiovascular symptoms including dyspnea, and 3) without known cardiovascular disease or self-reported diagnosis of myocardial infarction, stroke, or coronary revascularization. The study was approved by the Committee for the Protection of Human Subjects of the University of Texas Health Sciences Center at Houston. Written informed consent forms were obtained from all study participants.

### Measurements

#### Echocardiographic measurement and outcome measures

A standardized echocardiography ultrasound examination, including M-mode, 2-dimensional, Doppler velocity spectral waveform, color flow, and tissue Doppler, was performed using a Philips EPIQ scanner interfaced with a standard X5-1c MHz phased array probe. The examination was performed by an experienced registered diagnostic cardiac sonographer (EM). The echocardiographic images were read and interpreted by blinded expert readers (SL and SP) with advanced echocardiography training. Appropriate measurements were obtained, including left ventricular wall thickness, left ventricular mass index calculations, left atrial volumetric measurements, mitral inflow Doppler and tissue Doppler measurements including septal and lateral mitral annular displacements. Diastolic parameters were evaluated in accordance with the 2025 recommendations from the American Society of Echocardiography (10). Specifically, pulmonary artery systolic pressure and filling pressures using tissue Doppler imaging were ascertained. A total of 1,315 subjects were initially included but of these, 30 participants had a documented left ventricular ejection fraction of <50% and were therefore excluded from analysis (final sample size of 1,285 subjects).

### H_2_FPEF score

The H_2_FPEF score was initially derived using a cohort of patients with unexplained dyspnea who underwent invasive hemodynamic testing, and who had the diagnosis of HFpEF made based on elevated pulmonary capillary wedge pressure at rest (≥15 mmHg) or during exercise (≥25 mmHg). On multivariate analysis, factors found to be the strongest discriminators between HFpEF and non-cardiac etiologies of dyspnea were obesity [body mass index (BMI) > 30 kg/m^2^], atrial fibrillation, age >60 years, treatment with ≥2 antihypertensive drugs, and *E*/*e*′ > 9 and pulmonary artery systolic pressure >35 mm Hg by echocardiography (4). The H_2_FPEF score [H_2_ for Heavy and Hypertension, F for atrial Fibrillation, P for Pulmonary hypertension, E for Elderly, and F for Filling pressure] was then derived as a composite of these variables based on strength of association on logistic regression analysis, and subsequently validated with clinical outcomes data, particularly in outpatient settings (11–13).

**Figure 1:**
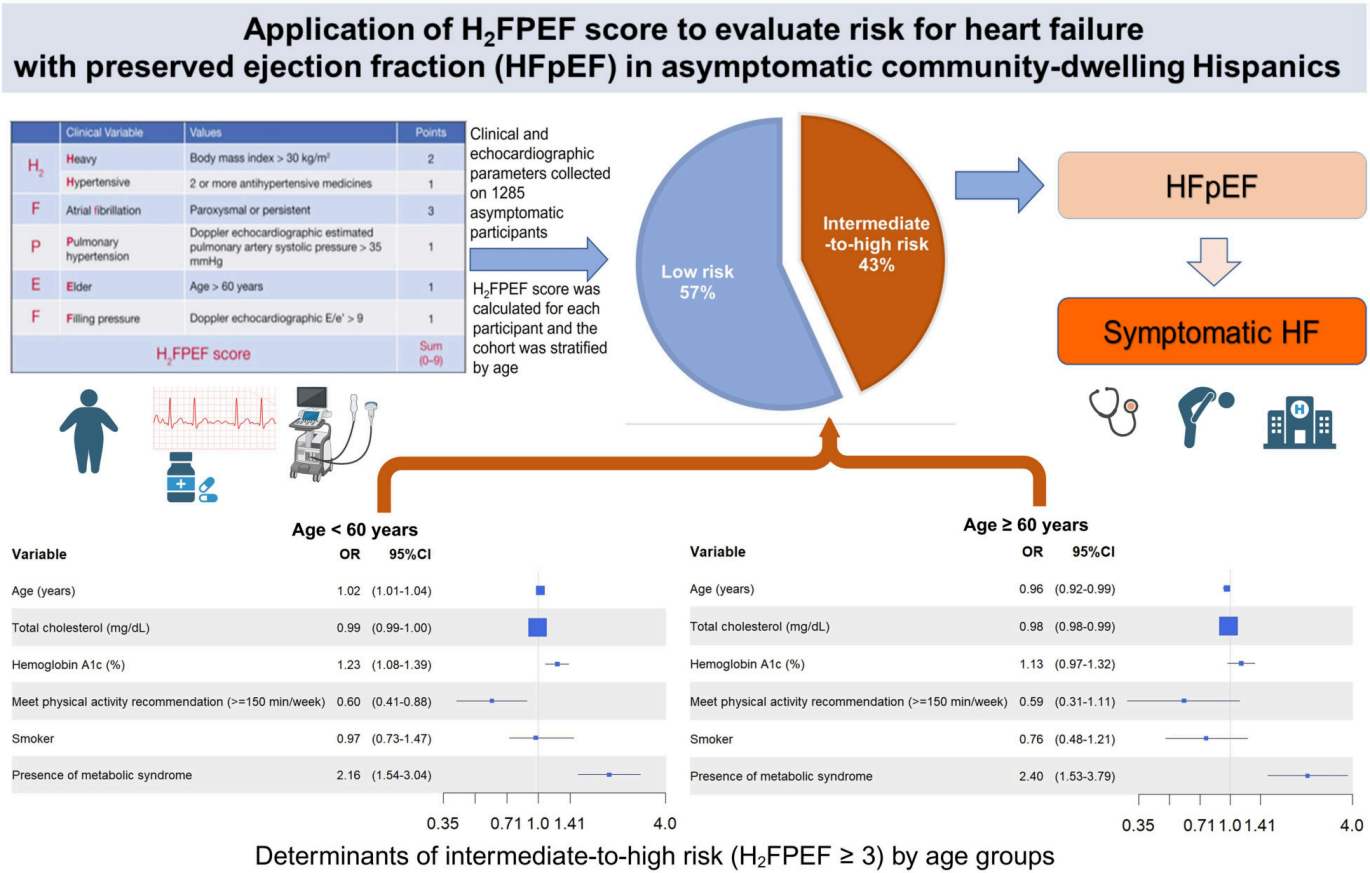
Application of H_2_FPEF score to evaluate risk for heart failure with preserved ejection fraction (HFpEF) in asymptomatic community-dwelling Hispanics.

A H_2_FPEF score was calculated for each subject in this cohort using the same criteria and weighting applied to the initial derivation cohort, with a range of 0–7 across the entire sample. Subjects were then classified as either low risk (H_2_FPEF score of 0–2) or intermediate-high risk (H_2_FPEF score of 3–7) for HFpEF (Figure 1).

### Statistical analysis

Descriptive statistics were summarized as mean and standard deviation (SD) for continuous variables and counts and percentages for categorical variables. To examine the effect modification of age on having a H_2_FPEF score of ≥3 (labeled as intermediate-high risk for HFpEF), the cohort was dichotomized into a younger group of <60 year-old and an older group of ≥60 year-old individuals. We used logistic regression models adjusted for demographic and cardiometabolic variables to estimate the odds of having an intermediate-high H_2_FPEF score among those <60 years and ≥60 years, separately and obtained *p* values from chi-square distribution using Wald test. Moreover, we ran sensitivity analyses using different age cutoff points (<65 years vs. ≥65 years), and we assessed for multicollinearity among model predictors using variance inflation factor estimates. Statistical significance was set at *p* < 0.05. All analyses were performed using R software (R version 4.3.3).

## Results

Demographic and metabolic characteristics are shown in Table 1. The average age was 52.9 ± 15.1 years, and 66.1% females. There was a high prevalence of metabolic risk factors in this cohort, with a high prevalence of overweight and obesity (84.4%) and a mean BMI of 31.0 ± 6.4 kg/m^2^. More than a third of the subjects (34.2%) were hypertensive, and more than a quarter of the patients (28.9%) have type 2 diabetes mellitus. Metabolic syndrome was found in 574 (44.7%) of the subjects.

**Table 1:** Demographic and clinical characteristics of the Cameron County Hispanic Cohort stratified by H_2_FPEF score.

Variable	Low risk H_2_FPEF score	Intermediate-high risk H_2_FPEF score	Overall Cohort	Overall missing	*P* value
	(*n* = 730)	(*N* = 555)	(*N* = 1,285)	(*N* %)	
Age (years)	49.1 (15.5)	58.0 (13.0)	52.9 (15.1)	0	<0.0001
BMI (kg/m^2^)	28.3 (5.14)	34.6 (6.07)	31.0 (6.37)	3 (0.2%)	<0.0001
Waist circumference (cm)	97.3 (13.2)	111 (13.6)	103 (15.1)	2 (0.2%)	<0.0001
Systolic BP (mmHg)	115 (16.4)	126 (19.6)	120 (18.6)	14 (1.1%)	<0.0001
Diastolic BP (mmHg)	70.6 (8.34)	74.0 (9.48)	72.1 (9.01)	14 (1.1%)	<0.0001
Mean FBG (mg/dL)	103 (35.5)	120 (47.7)	110 (42.1)	2 (0.2%)	<0.0001
Total cholesterol (mg/dL)	185 (38.0)	180 (40.8)	183 (39.2)	3 (0.2%)	0.04
Triglycerides (mg/dL)	143 (102)	159 (87.9)	150 (96.5)	2 (0.2%)	0.002
LDL (mg/dL)	108 (32.3)	101 (35.0)	105 (33.6)	15 (1.2%)	0.0007
HDL (mg/dL)	49.3 (13.0)	47.4 (12.6)	48.5 (12.8)	2 (0.2%)	0.008
HOMA-IR (mg/dL)	2.74 (2.35)	4.23 (3.63)	3.38 (3.05)	28 (2.2%)	<0.0001
Hemoglobin A1c (%)	5.97 (1.25)	6.67 (1.64)	6.27 (1.47)	2 (0.2%)	<0.0001
Current Smoker					
Yes	217 (29.7%)	174 (31.4%)	391 (30.4%)	22 (1.7%)	0.53
No	502 (68.8%)	370 (66.7%)	872 (67.9%)		
Years of Education					
≥High school diploma	376 (51.5%)	254 (45.8%)	630 (49.0%)	0	0.05
<High school diploma	354 (48.5%)	301 (54.2%)	655 (51.0%)		
Total minutes of exercise					
<150 min/week	476 (65.2%)	415 (74.8%)	891 (69.3%)	134 (10.4%)	<0.0001
≥150 min/week	187 (25.6%)	73 (13.2%)	260 (20.2%)		
Gender					
Male	256 (35.1%)	180 (32.4%)	436 (33.9%)	0	0.35
Female	474 (64.9%)	375 (67.6%)	849 (66.1%)		
Metabolic syndrome					
No	489 (67.0%)	222 (40.0%)	711 (55.3%)	0	<0.0001
Yes	241 (33.0%)	333 (60.0%)	574 (44.7%)		
BMI categories^a^					
Normal	180 (24.7%)	17 (3.1%)	197 (15.3%)	3 (0.2%)	<0.0001
Overweight	362 (49.6%)	51 (9.2%)	413 (32.1%)		
Obese	159 (21.8%)	418 (75.3%)	577 (44.9%)		
Morbid Obesity	27 (3.7%)	68 (12.3%)	94 (7.4%)		
Hypertension					
Yes	145 (19.9%)	295 (53.2%)	440 (34.2%)	26 (2.0%)	<0.0001
No	570 (78.1%)	249 (44.9%)	819 (63.7%)		
Hypercholesterolemia					
Yes	212 (29.0%)	245 (44.1%)	457 (35.6%)	26 (2.0%)	<0.0001
No	505 (69.2%)	297 (53.5%)	802 (62.4%)		
Type 2 DM					
No	594 (81.4%)	314 (56.6%)	908 (70.7%)	6 (0.5%)	<0.0001
Yes	133 (18.2%)	238 (42.9%)	371 (28.9%)		

BP, blood pressure; DM, diabetes mellitus; FBG, fasting blood glucose; LDL, low density lipoprotein; HDL, high density lipoprotein; HOMA-IR, homeostatic model assessment of insulin resistance test; BMI, body mass index;.

a BMI categories (26): Normal <25.0 kg/m^2^; Overweight ≥25.0 kg/m^2^, Obese ≥30 mg/m^2^, Morbid obesity ≥40 kg/m^2^.

In this community dwelling Hispanics/Latinos, 555 subjects (43.2%) were categorized as having intermediate-high H_2_FPEF score of 3–7. Among participants with intermediate-high H_2_FPEF score, the majority (*n* = 517; 93.2%) had elevated left ventricular filling pressure and/or pulmonary hypertension suggesting echocardiographic derangements in diastolic function. In young Hispanics with intermediate-high H_2_FPEF score in particular, only 3.2% (*n* = 9) had no echocardiographic criteria to score in the intermediate-high range (vs. 10.6% among those ≥ 60 years old). Univariate analysis showed that participants with intermediate-high H_2_FPEF score were about a decade older and had higher BMI and blood pressure, which are consistent with inclusion of these factors into the scoring algorithm. However, subjects with intermediate-high H_2_FPEF scores also had higher mean fasting glucose, higher homeostatic model assessment for insulin resistance (HOMA-IR) and hemoglobin A1c, had larger waist circumference, were more likely to be smokers, were more likely not to meet the recommended amount of total exercise per week, and were more likely to have hypercholesterolemia, diabetes, and the metabolic syndrome.

As HFpEF prevalence increases with age, we aimed to evaluate factors associated with higher H_2_FPEF score specifically in young Hispanics. We dichotomized the cohort to those <60 years of age (*n* = 852, mean age 45 ± 12 years) and those ≥ 60 years old (*n* = 433, mean age 68 ± 6 years). Table 2 demonstrates demographic and metabolic characteristics among young Mexican Americans aged <60 years, while Table 3 demonstrates these variables among those ≥60 years old. In young Mexican Americans, one third (*n* = 281, 33%) had intermediate-high H_2_FPEF scores, whereas among older Hispanics, the majority (*n* = 274, 63%) had intermediate-high H_2_FPEF scores. On multivariate analysis, for both age groups, age and presence of metabolic syndrome were significantly associated with an increased H_2_FPEF score. In younger participants only however, higher hemoglobin A1c (OR 1.23; 95% CI 1.08–1.39) and not meeting the recommended physical activity of ≥ 150 min of exercise per week (OR 0.60; 95% CI 0.41–0.88) were significantly associated with having an intermediate-high H_2_FPEF scores despite adjusting for covariates. There was lower total cholesterol in those with intermediate-high risk H_2_FPEF score, which likely reflects use of lipid lowering agents in this age group. Sensitivity analyses showed similar trends regardless of age cutoff points (i.e., at 60 years vs. 65 years), and no multicollinearity was detected in our models (Supplementary Tables S1 and S2).

**Table 2:** Multivariate analysis of factors associated with H_2_FPEF score for subjects <60 years (*n* = 852) in the Cameron County Hispanic Cohort.

Variable	Low risk H_2_FPEF Score^a^ Mean (SD) or N (%) *n* = 571	Intermediate-high risk H_2_FPEF score Mean (SD) or N (%) *n* = 281	Adjusted odds ratio (OR) [95% Confidence Interval]	*P* value
Age (years)	43.5 (12.4)	48.3 (10.5)	1.02 [1.01–1.04]	0.005*
Total cholesterol (mg/dL)	184 (38.3)	188 (41.1)	0.99 [0.99–1.00]	0.49
Hemoglobin A1c (%)	5.87 (1.15)	6.56 (1.71)	1.23 [1.08–1.39]	0.002*
Meet physical activity recommendation (≥150 min/week)	162 (28.4%)	47 (16.7%)	0.60 [0.41–0.88]	0.01*
Current Smoker	156 (27.3%)	83 (29.5%)	0.97 [0.73–1.47]	0.85
Presence of metabolic syndrome	180 (31.5%)	164 (58.4%)	2.16 [1.54–3.04]	<0.0001*

Low risk: H_2_FPEF score of 0–2; Intermediate-High risk: H_2_FPEF score of 3–7.

a Reference group.

* *p* < 0.05.

**Table 3:** Multivariate analysis of factors associated with H_2_FPEF score for subjects ≥60 years (*n* = 433) in the cameron county hispanic cohort.

Variable	Low risk H_2_FPEF score^a^ Mean (SD) or N (%) *n* = 159	Intermediate-high risk H_2_FPEF score Mean (SD) or N (%) *n* = 274	Adjusted odds ratio (OR) [95% Confidence Interval]	*P* value
Age (years)	69.0 (6.50)	67.9 (5.76)	0.96 [0.92–0.99]	0.04*
Total cholesterol (mg/dL)	189 (36.3)	172 (38.8)	0.98 [0.98–0.99]	0.0003*
Hemoglobin A1c (%)	6.34 (1.49)	6.78 (1.56)	1.13 [0.97–1.32]	0.13
Meet physical activity recommendation (≥150 min/week)	25 (15.7%)	26 (9.5%)	0.59 [0.31–1.11]	0.10
Current Smoker	61 (38.4%)	91 (33.2%)	0.76 [0.48–1.21]	0.25
Presence of metabolic syndrome	61 (38.4%)	169 (61.7%)	2.40 [1.53–3.79]	0.0002*

a Reference group; Low risk: H_2_FPEF score of 0–2; Intermediate-High risk: H_2_FPEF score of 3–7.

* *p* < 0.05.

## Discussion

This study shows that nearly half of asymptomatic community dwelling Hispanics in our cohort had intermediate-high H_2_FPEF scores. Especially concerning is that among those <60 years of age, a third already had intermediate-high H_2_FPEF scores. Our study also showed that the majority of participants with intermediate-high H_2_FPEF scores in our cohort met echocardiographic criteria of pulmonary hypertension and elevated filling pressures suggesting subclinical cardiac functional derangements already present in this relatively young Hispanic cohort. Age and the metabolic syndrome were significantly associated with intermediate-high H_2_FPEF scores in both those <60 and ≥60 years of age, however, only among younger participants were higher hemoglobin A1c and a sedentary lifestyle also significantly associated with intermediate-high H_2_FPEF scores. Other investigators have demonstrated the expanded use of the H_2_FPEF score, but to our knowledge, our study is the first to apply the H_2_FPEF score to a Hispanic cohort. Hence, our study adds to the existing literature regarding potential use of this algorithm for the detection of those at high risk for HFpEF in the outpatient setting, which may allow for earlier recognition of HFpEF or those at risk for HFpEF by general practitioners and cardiologists, particularly in communities with limited resources.

Our finding of high prevalence of intermediate-high H_2_FPEF scores is consistent with the documented underdiagnosis of HFpEF in the clinical setting. In the Echocardiographic Study of Latinos, almost half (49.7%) of middle aged or older Hispanics had some form of cardiac dysfunction (either systolic or diastolic dysfunction or both) and yet, only a minority (5.3%) of them were symptomatic or were clinically recognized as having HF (14). HFpEF constitutes nearly half of HF cases, with morbidity and mortality rates similar to those with HF with reduced ejection fractions (15). However, diagnosis remains challenging particularly in the early stages related in part to the lack of universally accepted diagnostic guidelines (16, 17).

Several algorithms and scoring systems have been proposed to aid in the diagnosis of HFpEF without the use of invasive hemodynamic testing. Two clinical scoring algorithms (HFA-PEFF and H_2_FPEF) have been described and validated in patients suspected of having HFpEF. The Heart Failure Association of the European Society of Cardiology proposed the HFA-PEFF score in 2019, which is a comprehensive multi-parametric scoring system to help in the diagnosis of HFpEF (18). The HFA-PEFF score includes major and minor criteria using echocardiographic or invasive hemodynamic measurements as well as brain natriuretic peptide levels. The H_2_FPEF score, on the other hand, was described in 2018 to help clinicians differentiate cardiac causes of dyspnea from non-cardiac causes, with validation performed in individuals with unexplained dyspnea who were referred for invasive hemodynamic exercise testing (4). Although both scores have proven useful in the diagnosis of unexplained dyspnea (13), H_2_FPEF is simpler to use without the need for obtaining a blood test. Since then, some investigators have applied the H_2_FPEF score to help ascertain HFpEF risk in the outpatient setting. In a Japanese prospective cohort study by Suzuki et al, the H_2_FPEF score was applied to stable patients followed in the outpatient clinic; the authors demonstrated that H_2_FPEF score was an independent predictor for future HF-related events including cardiovascular death and hospitalization for decompensated heart failure (mean follow up 517 days) (11). In a retrospective observational study, Sueta et al. also applied the H_2_FPEF score to consecutive HFpEF patients as a prognostic tool and demonstrated that a H_2_FPEF score cut off of 5.5 was a predictor for cardiovascular and HF-related events (mean follow up 50 months) (12). Furthermore, Selvaraj et al. demonstrated that in asymptomatic participants in the Atherosclerosis Risk in Communities (ARIC) Study (76.6% of the cohort), higher scores of both H_2_FPEF and HFA-PEF algorithms were associated with increased risk of incident HF hospitalization or death with a mean follow-up of 5.3 ± 1.2 years (13). Diagnosing HFpEF remains challenging in the outpatient setting and application of the H_2_FPEF score may improve recognition of these patients early in the disease continuum for initiation or intensification of life saving therapies.

In our study, age and the metabolic syndrome were significantly associated with increased odds of having intermediate-high risk H_2_FPEF scores, even after adjusting for covariates. However, in young Hispanics alone (< 60 years of age), both abnormal glucose control and a sedentary lifestyle were also significantly associated with a higher H_2_FPEF score. Prediabetes and diabetes induce structural and functional changes in the myocardium, including subclinical myocardial damage and a shift toward fatty acid oxidation (19, 20). Similarly, a sedentary lifestyle can also induce myocardial changes and lead to cardiac atrophy, decreased stroke volume, and pathologic cardiac remodeling (21, 22). Bankoski et al. showed that sedentary time was associated with the metabolic syndrome (23) and an inverse association between physical activity and risk of HFpEF has been demonstrated by Florido et al. among the participants of The ARIC Study (24). Using data from the American Heart Association's Get With The Guidelines-HF registry, Vivo et al. showed that among those hospitalized for HFpEF, more than a third (38.3%) of Hispanic patients were younger than 66 years (7). Hispanics also had a higher prevalence of diabetes, hypertension, and overweight/obesity compared to their non-Hispanic counterparts (7). A simple and useful tool such as the H_2_FPEF score to help practitioners identify persons at risk for HFpEF among Hispanics, particularly in communities with limited resources, may have implications for early initiation of preventive and life-saving therapies before transition to symptomatic HF.

Limitations of this study include its cross-sectional design and lack of longitudinal follow-up and lack of a comparison cohort, which precludes definitive conclusions about the long-term prognostic value of the H_2_FPEF score in predicting outcomes such as HF hospitalizations and death. Moreover, the high prevalence of our outcome (i.e., H_2_FPEF score) was likely to result in an inflated association between metabolic syndrome and H_2_FPEF score, as odds ratios tend to overestimate risk when the outcome is common. Although we excluded independent predictors from our models that are part of the H_2_FPEF score calculation (e.g., BMI), residual confounding may still exist. Furthermore, residual confounding from unmeasured confounders (e.g., medication use, sleep apnea, or sociodemographic disparities) might contribute to part or all of the observed association between metabolic syndrome and H_2_FPEF score. Another limitation is the relatively low prevalence of atrial fibrillation in our cohort. In the original study by Reddy et al (4)., the presence of atrial fibrillation conferred the highest odds ratio for HFpEF and was thus weighted the most heavily in calculation of the H_2_FPEF score (with a value of 3 points). Compared to the original validation cohort, however, where over one third of the subjects with HFpEF had atrial fibrillation, the prevalence of atrial fibrillation in our cohort was significantly lower, with only 2/1,285 subjects having documented atrial fibrillation. This is consistent, however, with a large study by Linares et al. that showed a low prevalence of atrial fibrillation among Mexican Americans (0.3%) when compared to other Hispanic sub-groups in the United States (25).

In summary, our study shows a high prevalence of intermediate-high H_2_FPEF scores in community dwelling Hispanics. Age and the metabolic syndrome were associated with high H_2_FPEF scores. However, among young Hispanics, higher hemoglobin A1c and a sedentary lifestyle were also independent predictors for having intermediate-high H_2_FPEF scores. In communities with limited resources such as the setting of the CCHC, there is decreased penetrance for guideline-recommended but expensive therapies such as sodium-glucose transport protein-2 inhibitors and glucagon-like peptide-1 agonists. Hence, early diagnosis and targeted prevention, focusing on cardiometabolic risk factors, including glucose control and exercise among young Hispanics, may mitigate this risk, leading to decreased incident clinical HF, and thus reduce the societal burden of this disease.

## Data Availability

The raw data supporting the conclusions of this article will be made available by the authors, without undue reservation.
