# Supplementary material for: HFpEF risk assessment using H2FPEF score in community-dwelling young Hispanic adults
Source: Front Cardiovasc Med. 2026 May 29;13:1832145. doi: 10.3389/fcvm.2026.1832145 (PMC13262497; doi:10.3389/fcvm.2026.1832145)
Supplement: Supplementary file 1 [file Datasheet1.docx]

Supplemental table S1. Multicollinearity check using variance inflation factors (VIF).

| **Variable** | **VIF from <60 model** | **VIF from >=60 model** |
| --- | --- | --- |
| Age (years) | 1.15 | 1.07 |
| Total cholesterol (mg/dL) | 1.07 | 1.05 |
| Hemoglobin A1c (%) | 1.15 | 1.04 |
| Meet physical activity recommendation (>=150 min/week) | 1.02 | 1.03 |
| Smoker | 1.01 | 1.01 |
| Presence of metabolic syndrome | 1.14 | 1.04 |

Supplemental table S2. Sensitivity analysis using multivariate logistic regression with age cutoff at 65 years.

| **Variable** | **<65 years** | | **≥65 years** | |
| --- | --- | --- | --- | --- |
|  | **Adjusted Odds Ratio (OR)**  [95% Confidence Interval] | **P value** | **Adjusted Odds Ratio (OR)**  [95% Confidence Interval] | **P value** |
| Age (years) | 1.04 [1.02-1.05] | <0.0001* | 0.96 [0.91-1.01] | 0.08 |
| Total cholesterol (mg/dL) | 0.99 [0.99-1.00] | 0.08 | 0.98 [0.98-0.99] | 0.002* |
| Hemoglobin A1c (%) | 1.17 [1.05-1.31] | 0.005* | 1.24 [1.01-1.55] | 0.04* |
| Meet physical activity recommendation (>=150 min/week) | 0.59 [0.41-0.84] | 0.004* | 0.71 [0.30-1.66] | 0.42 |
| Smoker | 0.99 [0.72-1.37] | 0.95 | 0.75 [0.43-1.30] | 0.30 |
| Presence of metabolic syndrome | 2.29 [1.68-3.13] | <0.0001* | 1.91 [1.12-3.28] | 0.02* |
